# Presenilin 1 phosphorylation regulates amyloid-β degradation by microglia

**DOI:** 10.1038/s41380-020-0856-8

**Published:** 2020-08-13

**Authors:** Jose Henrique Ledo, Thomas Liebmann, Ran Zhang, Jerry C. Chang, Estefania P. Azevedo, Eitan Wong, Hernandez Moura Silva, Olga G. Troyanskaya, Victor Bustos, Paul Greengard

**Affiliations:** 1 Laboratory of Molecular and Cellular Neuroscience, The Rockefeller University, New York, NY 10065, USA; 2 Lewis Sigler Institute for Integrative Genomics, Princeton University, Princeton, NJ 08544, USA; 3 Laboratory of Molecular Genetics, The Rockefeller University, New York, NY 10065, USA; 4 Chemical Biology Program, Memorial Sloan Kettering Cancer Center, New York, NY 10065, USA; 5 Skirball Institute of Biomolecular Medicine, New York University School of Medicine, New York, NY 10016, USA; 6 Flatiron Institute, Simons Foundation, New York, NY 10010, USA

## Abstract

Amyloid-β peptide (Aβ) accumulation in the brain is a hallmark of Alzheimer’s Disease. An important mechanism of Aβ clearance in the brain is uptake and degradation by microglia. Presenilin 1 (PS1) is the catalytic subunit of γ-secretase, an enzyme complex responsible for the maturation of multiple substrates, such as Aβ. Although PS1 has been extensively studied in neurons, the role of PS1 in microglia is incompletely understood. Here we report that microglia containing phospho-deficient mutant PS1 display a slower kinetic response to micro injury in the brain in vivo and the inability to degrade Aβ oligomers due to a phagolysosome dysfunction. An Alzheimer’s mouse model containing phospho-deficient PS1 show severe Aβ accumulation in microglia as well as the postsynaptic protein PSD95. Our results demonstrate a novel mechanism by which PS1 modulates microglial function and contributes to Alzheimer’s -associated phenotypes.

## Introduction

Microglia are yolk sac–derived innate immune cells in the brain that play key roles in multiple stages of Alzheimer’s disease (AD), from inflammatory responses and synapse pruning to degradation of amyloid-β (Aβ) peptide, all of which are thought to drive AD pathology progression [1, 2]. For example, in early stages of AD, microglia actively internalize different assemblies of Aβ and eliminate them through endolysosomal degradation [3–5]. Aβ clearance by microglia may be inhibited in late AD due to accumulation of plaques. The buildup of Aβ further leads to the release of pro-inflammatory cytokines, thereby increasing neurotoxicity in AD brains [1, 3, 6]. Moreover, recent genome-wide association studies of sporadic AD have identified several genes linked to microglia function, such as *Trem2* and *Cd33* [7–9]. TREM2 facilitates Aβ phagocytosis by microglia [10] and CD33 impairs microglia-mediated Aβ clearance [8]. However, the mechanisms by which microglial cells become defective in AD is incompletely understood.

Presenilin 1 *(Psen1* gene—PS1 protein) is the catalytic subunit of γ-secretase, an enzyme complex responsible for the cleavage of multiple substrates [11], including the amyloid precursor protein (APP). The sequential cleavage of APP by β- and γ-secretase leads to the Aβ production, which is thought to play a causative role in Alzheimer’s pathogenesis [12]. Although PS1 is broadly expressed in the central nervous system of humans, within the mouse brain cortex it is highly expressed in microglial cells [13]. Curiously, PS1 has been mostly studied in neurons. Early reports have shown that γ-secretase plays a significant role in microglia [14]. For example, microglial activation is prevented by γ-secretase inhibitors in a model of focal ischemic stroke [15]. In mouse microglial cell lines, γ‐secretase inhibitors attenuated pro‐inflammatory cytokine expression upon LPS treatment [16, 17]. Data also suggest that a familial Alzheimer’s disease (FAD) mutation in PS1 leads microglia to become more reactive to pro-inflammatory stimuli [14, 18]. Data implicating a significant role for PS1 in microglia are mostly focused on inflammation. The extent to which PS1 significantly contribute to microglial function and development of AD pathological hallmarks beyond inflammation is not well understood.

Phosphorylation is the most common mechanism of regulating protein function and cellular signal transduction. Curiously, PS1 phosphorylation seems to have little impact on γ-secretase activity [19]; however, PS1 phosphorylation at serine 367 regulates important biological processes, some of which related to Aβ metabolism and AD [20, 21]. We have recently generated and characterized a knock-in mouse expressing a phospho-deficient PS1 (*Psen1*^*S367A/S367A*^, denoted *Psen1*^*KI/KI*^), which, when crossed with an AD mouse model (J20—mouse expressing human APP bearing Swedish and Indiana AD-causing mutations), exhibits a significant accumulation of Aβ independent of γ-secretase activity [20, 21]. This increase was shown to be due to decreased conversion of βCTF to Aβ, through autophagy-mediated βCTF degradation [20, 21]. The present study adds an additional mechanism to our previous findings in neurons, we show that PS1 phosphorylation at serine 367 plays an important role in regulating Aβ levels by microglia. Live brain imaging in the adult mouse revealed that microglial cells from *Psen1*^*KI/KI*^ mice have decreased ability to respond to brain injury. Combining a systematic profile of microglia transcripts with experiments in primary cell cultures, we show that microglia from *Psen1*^*KI/KI*^ mice displayed abnormal autophagy-lysosomal pathway and inability to degrade Aβ. In addition, we found a significant accumulation of Aβ and the post-synaptic marker PSD95 in *Psen1*^*KI/KI*^ microglia in an Alzheimer’s mouse model. Collectively, our data show that the PS1 plays an important role in microglial cells which is associated to disease-related phenotypes.

## Results

### *Psen1*^*KI/KI*^ microglia show abnormal response to a laser-induced micro injury in vivo

**Fig. 1:**
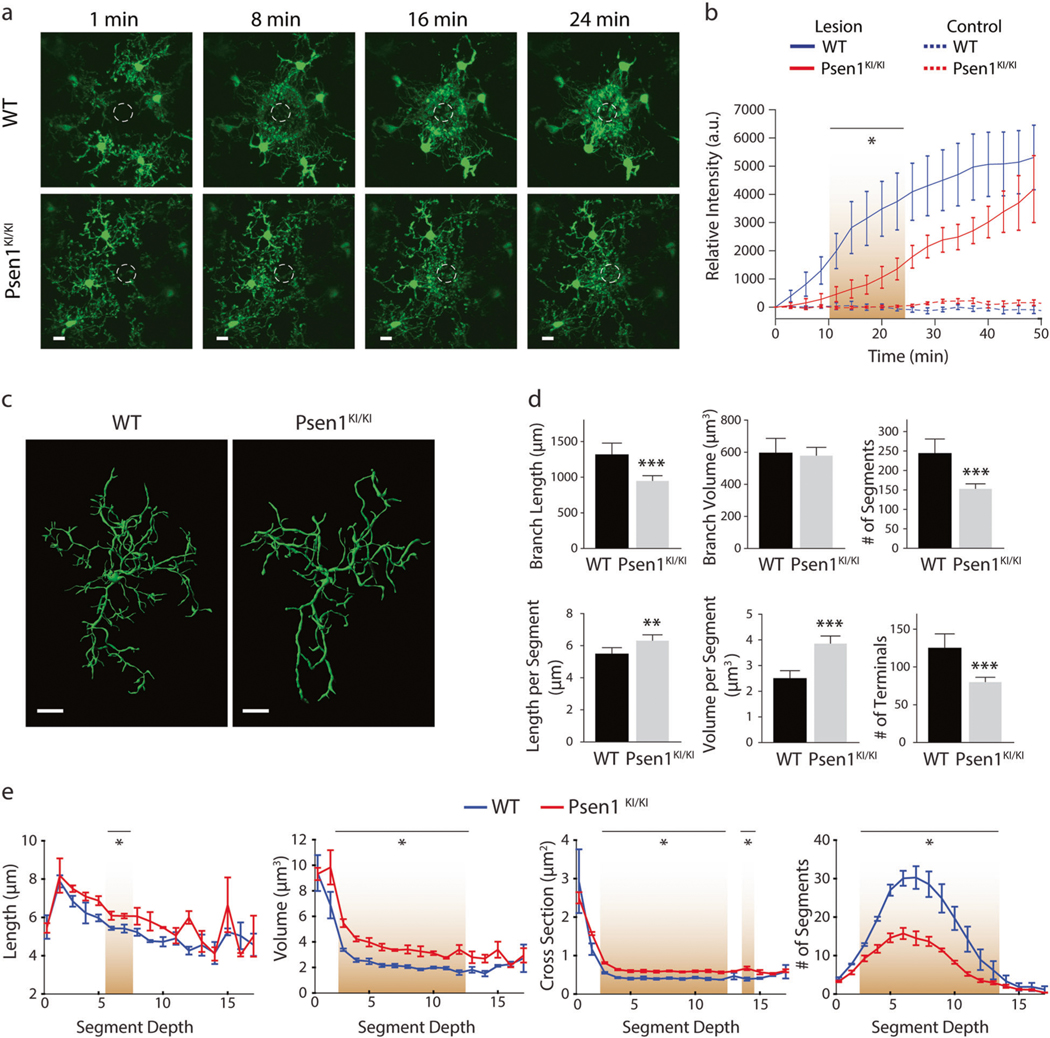
*Psen1*^*KI/KI*^ alters microglial morphology and function in vivo. **a** Representative time-lapse response of microglia to laser injury in vivo. Volume stacks of microglia in the CX3CR1-EGFP mice are visualized through a cranial window with multi photon imaging immediately following a focused laser injury (indicated by a dashed white circle). Scale bar represent 10 μm **b** Time profile of microglia branch encroachment into injury region. **c** Representative 3D filament tracings of entire microglia in the cortex of WT and *Psen1*^*KI/KI*^ mouse brains after Iba1 labeling and high-resolution confocal stack imaging. Scale bars represent 10 μm **d** Characterization of microglia branching: total branch length, total branch volume, total number of segments (between junction points), segment length, segment volume, number of terminal branches. Total volume refers to total volume of all branches of single microglia, it does not include cell body (schematic diagram Supplementary Fig. 1c). **e** Average length, volume, cross sectional area and frequency of segments at the given branch depth from the soma. Brown gradients represent points where statistical significance was observed. Bar and line plots represent means ± sem. *p < 0.05, two sample *t* test. For in vivo experiments, *N* **=** 5–7 mice per group. For microglia morphology, *N* = 10 microglia per mouse, three different mice for each genotype. Blind analyses were performed.

In the adult brain, ramified and mobile processes of microglia continuously scan the parenchyma and efficiently respond to any local disturbances [22]. Upon brain injury, local microglia respond rapidly by altering their morphology and behavior to a highly mobile, migratory phenotype [22, 23]. To evaluate functional alterations in microglia due to the lack of PS1 phosphorylation at serine 367, we measured microglial response to a local brain injury by performing in vivo recording in the live mouse brain using a glass-covered cranial window preparation combined with 2-photon in vivo microscopy [24]. To target PS1 and visualize microglia, we crossed a phospho-deficient PS1 mouse (PS1 S367A, *Psen1*^*KI/KI*^ (Supplementary Fig. S1a, b)), with fractalkine receptor CX3CR1-EGFP knock-in mouse [25]. Upon laser micro-injury approximately 65 μm below the mouse cortex surface, microglia from *Psen1*^*KI/KI*^ mice displayed a slower kinetic response compared to WT counterparts (Fig. 1a, b; Supplementary Movie 1). These data showed that the response to a brain injury, an elementary function of microglia, is altered in *Psen1*^*KI/KI*^ compared to control microglia. Microglia with disrupted morphology, such as number or length of ramifications, show an impaired ability to efficiently survey the local environment [23]. Therefore, we next characterized microglial morphology in mice. For this purpose, we imaged the cerebral cortex and traced Iba1^+^-stained microglia in uninjured mice (Fig. 1c; Supplementary Movie 2). We found that microglia branch length, the number of branch segments; segment length, segment volume and number of terminals were significantly altered in *Psen1*^*KI/KI*^ microglia whereas the total volume was not different between groups (Fig. 1c–e; Supplementary Fig. S1c). High mobility and a complex arborization allow microglial cells to efficiently survey the brain parenchyma, whereas defects in this process can compromise how microglia respond to external signaling to activate phagocytosis. In addition to microglial morphology we also investigated microglial number in WT and *Psen1*^*KI/KI*^ mice (Supplementary Fig. S1d). Our data showed that microglial number in *Psen1*^*KI/KI*^ mice is equivalent to WT mice. Collectively, our results demonstrate that the lack of phosphorylation of PS1 at serine 367 disrupts an elementary microglial response to a brain injury in vivo.

### Gene expression profiles of WT and *Psen1* ^*KI/KI*^ microglia

**Fig. 2:**
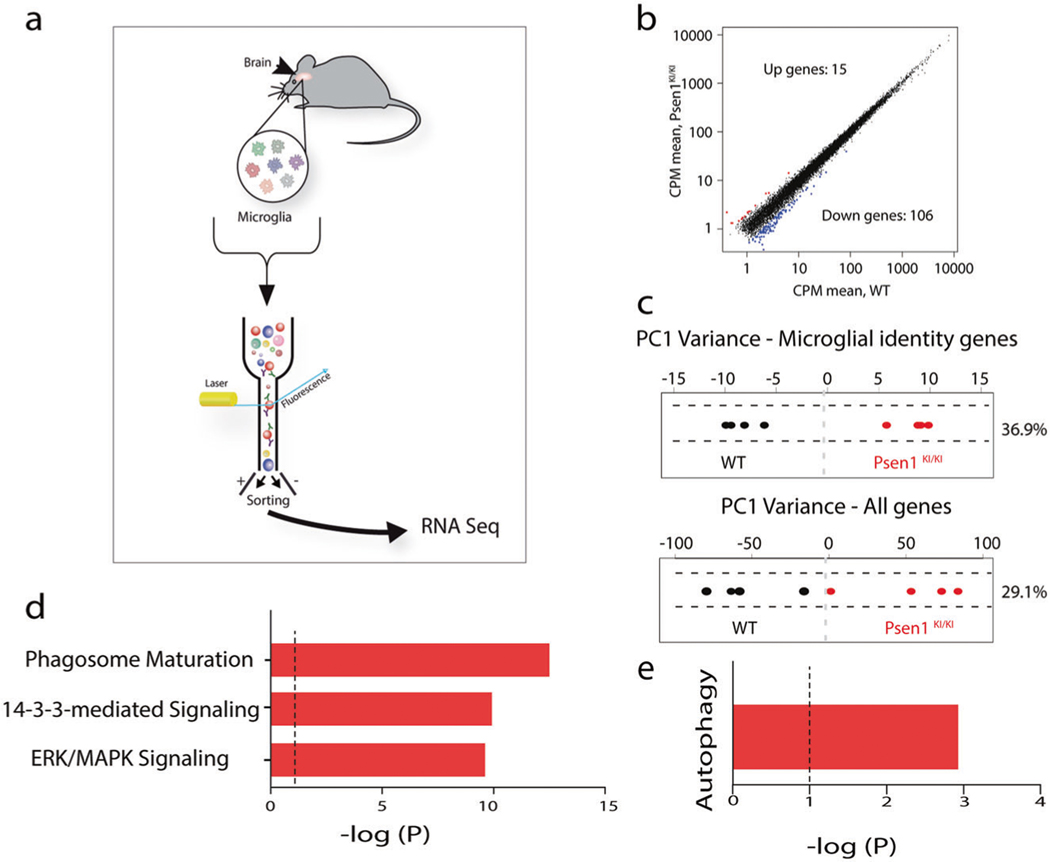
Gene-expression patterns of WT and *Psen1*^*KI/KI*^ microglia. RNA sequencing performed in adult microglia from WT and *Psen1*^*KI/KI*^ mouse. **a** Schematic diagram showing the strategy used to profile microglial transcripts. **b** Expression (Counts per million, CPM) of WT plotted against CPM *Psen1*^*KI/KI*^ microglia. **c** Principal component analysis restricted to microglia identity genes expressed in WT or *Psen1*^*KI/KI*^ adult microglia (upper panel) or all genes (lower panel). **d, e** Biological pathways terms enriched in genes differentially expressed in Psen1^KI/KI^ microglia compared to WT microglia identified by Ingenuity Pathway Analysis (IPA). *N* **=** 4 mice per group.

We next assessed microglial gene expression profiles in WT and *Psen1*^*KI/KI*^ mice to comprehensively evaluate biological pathways differentially regulated in *Psen1*^*KI/KI*^ (Fig. 2a; Supplementary Fig. S1e). Analysis of adult control WT and *Psen1*^*KI/KI*^ microglia revealed 121 genes differentially expressed in *Psen1*^*KI/KI*^ microglia (Fig. 2b). Interestingly, examination of transcriptional patterns of WT and *Psen1*^*KI/KI*^ microglia using PCA analysis, suggests that WT and *Psen1*^*KI/KI*^ microglia are distinct populations (Fig. 2c). This result holds when analyzing expression of previously defined microglia identity genes [26] (Fig. 2c, upper panel) or all genes (Fig. 2c, lower panel). We next performed pathway analysis using Ingenuity Pathway Analysis (IPA). The most significantly altered pathway elicited in *Psen1*^*KI/KI*^ microglia was related to phagosome maturation (−log *p* 12.5) (Fig. 2d). Previous reports have shown that phagosome formation at the tip of microglia branches is critical for the engulfment of damaged neurons and cell debris by microglia, such process is exacerbated upon brain microinjury [23, 27]. Defects in phagosome formation or processing capacity is potentially related to the slower kinetic response of *Psen1*^*KI/KI*^ microglia to a micro injury in the brain. Our analysis also revealed that the 14–3-3 pathway was altered in *Psen1*^*KI/KI*^ microglia (−log *p* 9.92), which represent conserved regulatory proteins that act in a variety of cellular processes [28]. Another pathway significantly affected by *Psen1*^*KI/KI*^ was the ERK/MAPK signaling pathway (−log *p* 9.61) which has been shown to be specifically activated in microglia in a pre-clinical model of AD pathology and human post-mortem AD brains [29]. Interestingly, ERK inhibition reduces the ability of microglia to phagocytose Aβ42 [29]. In addition, our analysis revealed that the autophagy pathway was significantly affected by *Psen1*^*KI/KI*^ in microglia (−log *p* 2.93) (Fig. 2e). These data showed that *Psen1*^*KI/KI*^ disturbed important microglial pathways, of which the most significant changes are related to autophagy. Thus, we next sought to investigate autophagosomes related structures in microglia from WT and *Psen1*^*KI/KI*^ mice.

### *Psen1*^*KI/KI*^ disrupts the autophagy-lysosomal pathway in microglia

**Fig. 3:**
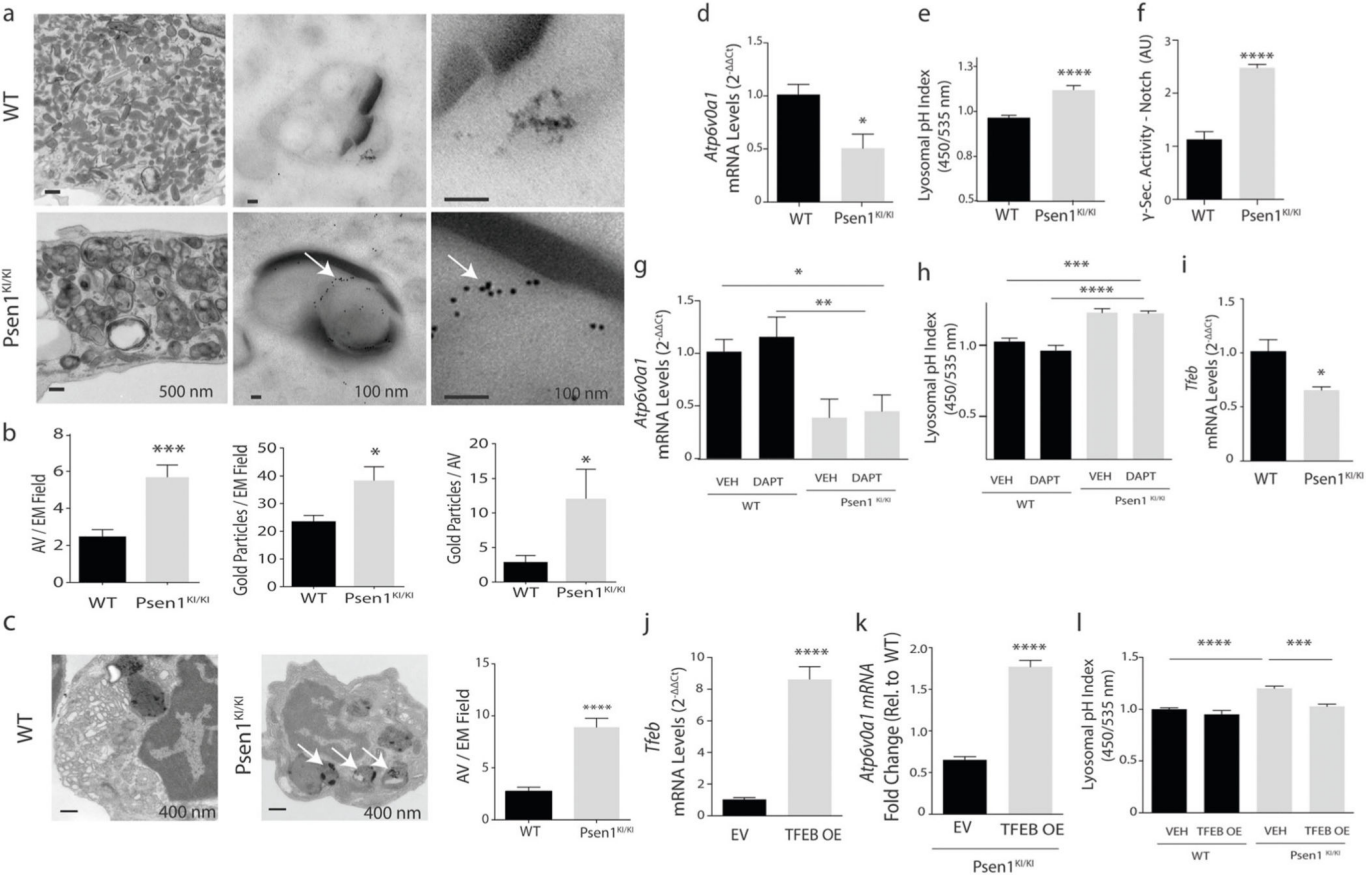
*Psen1*^*KI/KI*^ disrupts autophagy-lysosomal pathway in microglia. **a** Electron microscopy images of autophagosome vacuoles or immunolabeling of LC3-GFP on primary microglia cultures from WT or *Psen1*^*KI/KI*^ mice. **b** Quantification of autophagosome vacuoles per EM field, gold particles (LC3-GFP) per EM field and gold particles (LC3-GFP) per autophagosome vacuoles. **c** Electron microscopy images of autophagosome vacuoles on microglia sorted from WT or *Psen1*^*KI/KI*^ adult mice and quantification of autophagosome vacuoles per EM field and number of autophagosomes vacuoles according to their size (left panel). 16 EM fields per group (genotype), three independent microglia cultures from three different mice per group. **d** Quantitative RT-PCR of *Atp6v0a1* in WT or *Psen1*^*KI/KI*^ primary microglia cell cultures **e** Lysosomal pH of primary microglia cultures from WT or *Psen1*^*KI/KI*^ mice was determined using LysoSensor Yellow/Blue dextran (450/535 nm) **f** Exo-cell γ-secretase activity assay for recombinant Notch substrate in WT or Psen1^*KI/KI*^ microglial cells. γ-Secretase activity is expressed as arbitrary units (for details see “Methods”) **g** Quantitative RT-PCR of *Atp6v0a1* in WT or *Psen1*^*KI/KI*^ primary microglia cell cultures treated with vehicle or DAPT (10 μM) for 24 h. **h** Lysosomal pH of primary microglia cultures from WT or *Psen1*^*KI/KI*^ mice treated with vehicle or DAPT (10 μM) for 24 h. **i** Quantitative RT-PCR of TFEB in WT or *Psen1*^KI/KI^ primary microglia cell cultures **j** Microglial cells were transduced with an empty vector or TFEB-expressing lentivirus, and TFEB mRNA levels was assessed by qPCR. EV **=** empty vector, OE **=** overexpression. **k** Quantitative RT-PCR of *Atp6v0a1* in Psen1^*KI/KI*^ microglial cell cultures infected with an empty vector or TFEB-expressing lentivirus. **l** Lysosomal pH of primary microglia cultures transduced with an empty vector or TFEB-expressing lentivirus. Bar plots represent means ± sem. *p < 0.05, **p < 0.01, ***p < 0.001, ****p < 0.0001, two sample Student’s *t* test or one-way ANOVA followed by Duncan’s Method. *N* **=** 5 biological replicates from independent microglia cultures.

Using primary microglial cell cultures, we observed that *Psen1*^*KI/KI*^-derived microglia accumulated autophagosome vacuoles (AVs), identified based on their size and morphology, containing excessive undigested material and LC3 protein (Fig. 3a, b, white arrows indicate LC3), which suggests abnormalities in autophagy. Next, we asked whether abnormal AV accumulation found in vitro would be present in the mouse brain microglia. To investigate that, we sorted microglia from WT and *Psen1*^*KI/KI*^ adult mice and performed electron microscopy. Results showed that mice *Psen1*^*KI/KI*^ microglia have a much higher number of AVs containing excessive undigested material than WT microglia (Fig. 3c, white arrows). Acidification is an essential step during phagosome maturation which critically depends on the activity of the V-type (vacuolar) ATPases, proton pumps [30]. Interestingly, our RNA-seq data also revealed that the expression of the lysosomal V-ATPase *Atp6v0a1* was decreased in *Psen1*^*KI/KI*^ microglia (Supplementary Table S1). We further confirmed this data by qPCR (Fig. 3d). Since V-ATPases play a major role in the acidification of intracellular organelles such as lysosomes, we asked whether *Psen1*^*KI/KI*^ microglia present different pH values from WT microglia. The data showed that *Psen1*^*KI/KI*^ microglia have higher pH values than WT microglia (Fig. 3e). These data suggest that the autophagy–lysosomal pathway in microglia is disrupted by the lack of phosphorylation of PS1 at serine 367. We did not find differences in *Atp6v0a1* mRNA or lysosomal pH in WT microglia compared to *Psen1* KO (Supplementary Fig. 1f), similar to Zhang et al. [31]. One of the most thoroughly studied substrates of γ-secretase in addition to APP is Notch. Interestingly, autophagy and endocytic pathways regulate Notch degradation, signaling and, importantly, Notch intracellular domain cleavage [32–34]. Although our previous report showed that γ-secretase activity toward APP is not affected by *Psen1*^*KI/KI*^, it is unknown the impact of *Psen1*^*KI/K*^ in γ-secretase activity toward Notch in microglial cells. Thus, we next investigated γ-secretase activity toward Notch in microglial cell cultures of WT and *Psen1*^*KI/KI*^ mice. Our data revealed a robust increase in γ-secretase activity toward Notch in *Psen1*^*KI/KI*^ microglia compared to WT (Fig. 3f). This result prompted us to investigate whether the reduced expression of the lysosomal V-ATPase *Atp6v0a1* caused by *Psen1*^*KI/KI*^ was downstream to Notch cleavage. Pharmacological inhibition of γ-secretase activity in microglial cells did not change *Atp6v0a1* mRNA or lysosomal pH in *Psen1*^*KI/KI*^ or WT microglia (Fig. 3g, h). Previous reports have shown that *Psen1* deficiency suppress autophagy independent of γ-secretase activity, and cells deficient of *Psen1* have decreased levels of the lysosomal ATPase V-ATP6v and the transcriptional factor EB (TFEB) [35, 36], a master regulator of lysosome biogenesis and autophagy [37]. Interestingly, TFEB overexpression has been shown to increase the levels of v-ATP6v0a1 [38]. We, thus, explored whether decreased lysosomal *Atp6v0a1* mRNA and increased lysosomal pH in *Psen1*^*KI/KI*^ microglia was mediated by TFEB. Our RNA sequencing data from microglia sorted from mice brain (Supplementary Table S1) and qPCR (Fig. 3i) showed that TFEB mRNA level was decreased in *Psen1*^*KI/KI*^ microglial cells compared to WT microglia. We therefore sought to overexpress an active form TFEB in WT and *Psen1*^*KI/KI*^ microglia (Fig. 3j) and evaluate *ATP6v0a1* mRNA level and lysosomal pH (Fig. 3k, l). Our data showed that the active form of TFEB in *Psen1*^*KI/KI*^ microglia increased *ATP6v0a1* mRNA level and normalized lysosomal pH compared to WT microglia (Fig. 3k, l).

### *Psen1*^*KI/KI*^ increases Aβ accumulation in microglial cells in an Alzheimer’s mouse model

**Fig. 4:**
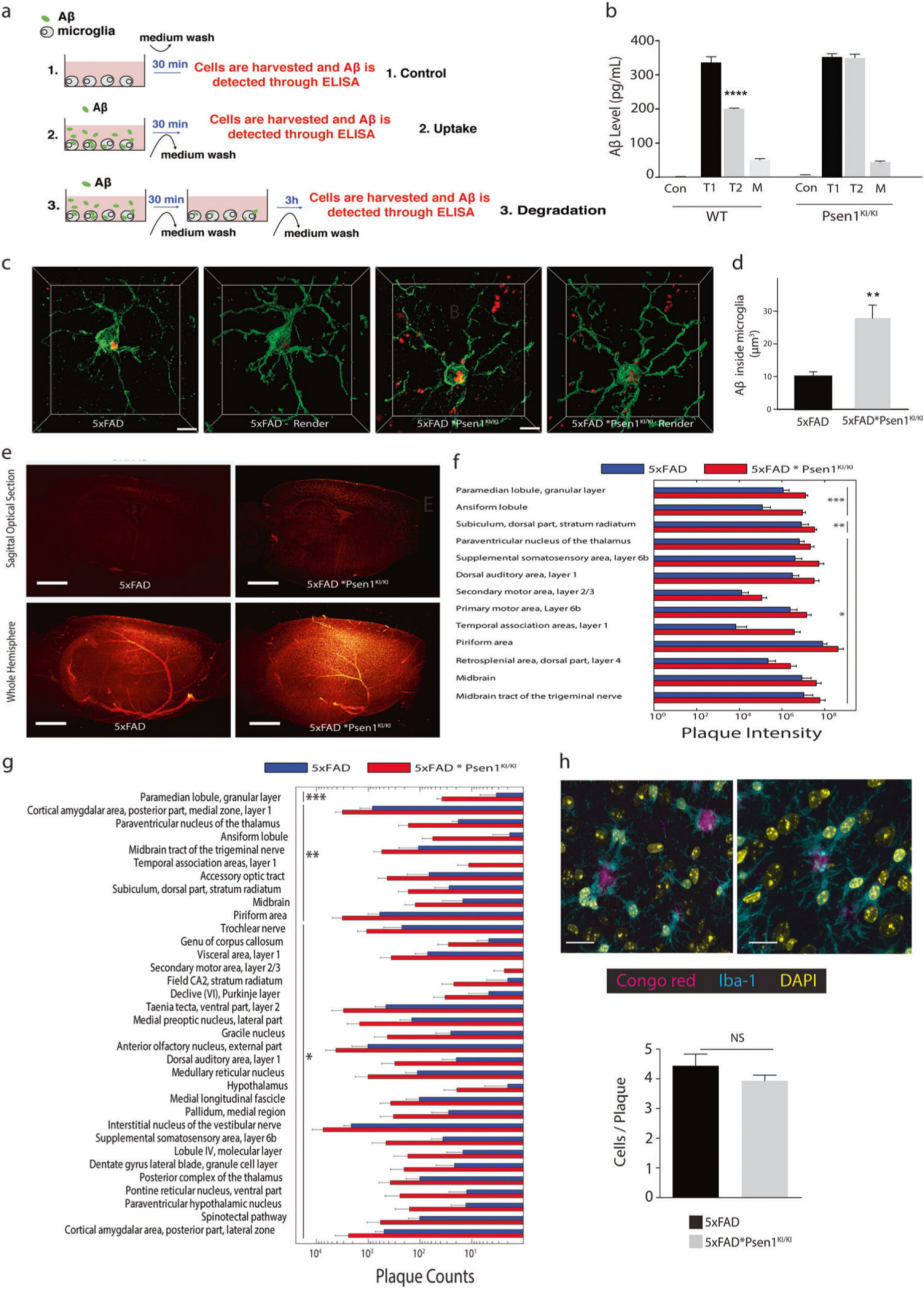
*Psen1*^*KI/KI*^ increases Aβ accumulation in microglia in an Alzheimer’s mouse model. **a** Schematic diagram of Aβ uptake and degradation assay. **b** Primary microglia cultures derived from WT and *Psen1*^*KI/KI*^ mice were treated with 500 nM of Aβ oligomers (Aβ 1–42) for 30 min (*T* **=** 1). After incubation, intracellular Aβ oligomers were detected by ELISA. For degradation, after initial incubation with Aβ oligomers, cells were washed and incubated for an additional 3 h (*T* **=** 2). Con **=** control, cells only. M **=** medium, Aβ oligomers detection in the cell culture medium after degradation. **c** Representative micrographs of 3D immunohistochemical distribution and surface-rendered reconstruction of microglia (green) and Aβ (red) in 50-μm thick cryosections from 5xFAD and 5xFAD * *Psen1*^*KI/KI*^ mice. Scale bar, 5 μm. **d** Calculation of Aβ internalization in microglia of 5xFAD and 5xFAD * *Psen1*^*KI/KI*^ mice. **e** Aβ plaque staining in cleared 3-month-old 5xFAD or 5xFAD*Psen1^*KI/KI*^ mouse brain hemispheres visualized with light sheet microscopy. Images are 100 μm sagittal optical sections and whole hemisphere maximum intensity projections, respectively. Scale bars represent 1 mm. **f** Aβ plaque intensity and **g** Aβ plaque count in different areas of the brain determined with ClearMap. A table of all detection values is available in Supplementary Table S1. **h** Representative confocal images of Iba1 immunolabelled microglia (cyan) around Congo red-labeled amyloid plaques (magenta) in a 3-month-old 5xFAD (left panel) and 5xFAD*Psen1^*KI/KI*^ (right panel) mice. Microglia coverage was quantified as number of microglia in contact with each plaque. *N* > 90 plaques (1–20 μm in diameter). Bar plots represent means ± sem. **p < 0.01, ***p < 0.001, Student’s *t* test or one-way ANOVA followed by Duncan’s Method. *N* **=** 5 biological replicates from independent microglia cultures. *N* **=** 5 mice per group.

The autophagy-lysosomal pathway has been implicated in neurodegenerative diseases, including AD [39]. Experimental evidences have shown that autophagy plays an important role in Aβ metabolism [20, 40–44]. Thus, we next challenged microglial cells with soluble Aβ oligomers to evaluate uptake and degradation of Aβ in WT and *Psen1*^*KI/KI*^ microglia (Fig. 4a). While *Psen1*^*KI/KI*^-derived microglia showed Aβ oligomer uptake rates similar to those seen in WT microglia, they displayed an impaired capacity for Aβ oligomer degradation (Fig. 4b).

An interplay between production and degradation dictate Aβ levels in the brain. Microglial cells contribute significantly to Aβ removal through degradation of the soluble fraction, and defects in Aβ clearance have been shown to accelerate AD pathology [44, 45]. Since wild-type mice do not develop age-associated amyloid pathology, we next crossed *Psen1*^*KI/KI*^ mice with 5×FAD mice, an accelerated model of AD that displays severe amyloid pathology [46] (5xFAD * *Psen1*^*KI/KI*^ mouse was hemizygous for FAD mutations and homozygous for *Psen1*^*KI/KI*^.) To evaluate the impact of *Psen1*^*KI/KI*^ on microglial Aβ internalization in mouse brain, we measured Aβ accumulation in microglia in the brain of 5xFAD and 5xFAD * *Psen1*^*KI/KI*^ mice. Confocal imaging of Aβ and Iba1 in brain tissue sections revealed that more Aβ was retained within 5xFAD * *Psen1*^*KI/KI*^ microglia compared to the 5xFAD (Fig. 4c, d). To comprehensively assess the impact of *Psen1*^*KI/KI*^ on Aβ accumulation in the entire brain, we applied the tissue clearing method iDISCO on intact brain hemispheres stained with Congo red (Fig. 4e; Supplementary Movie 3). Quantification of Aβ plaques, performed with region-specificity using ClearMap [47] revealed that 3 month old 5xFAD * *Psen1*^*KI/KI*^ mice accumulate significantly more Aβ plaques in 35 different areas of the brain than age-matched 5xFAD control mice (Fig. 4f, g). Of note, 5xFAD * *Psen1*^*KI/KI*^ mice not only accumulated more Aβ plaques but also soluble Aβ 40 and 42 compared to 5xFAD mice (Supplementary Fig. S2a). Our previous report showed that *Psen1*^*KI/KI*^ does not increase Aβ production through increased activity of γ-secretase toward APP [21]. To confirm that increased Aβ levels in 5xFAD * *Psen1*^*KI/KI*^ mice brains were not due to an increase in Aβ production (e.g., increased γ-secretase activity toward APP), we performed γ-secretase activity in 5xFAD and 5xFAD * *Psen1*^*KI/KI*^ mice brains (Supplementary Fig. S2b). Our data showed no difference in γ-secretase activity toward APP in 5xFAD * *Psen1*^*KI/KI*^ mice compared to 5xFAD. Thus, dismissing the possibility that increased Aβ accumulation inside microglia in 5xFAD * *Psen1*^*KI/KI*^ mouse brain resulted from a general increase in Aβ production. Due to the fact that Aβplaques are tightly enclosed by microglia processes and constitute a barrier that is critical for limiting plaque affinity for the neurotoxic soluble Aβ42 [48], we next evaluated the number of microglial cells associated with the enveloping of Aβ plaques. We found that the number of microglia associated with the enveloping of plaques in 5xFAD * *Psen1*^*KI/KI*^ mice is comparable to 5xFAD mice (Fig. 4h), suggesting that the increase in Aβ in 5xFAD * *Psen1*^*KI/KI*^ mice was not due to reduced number of microglia interacting with Aβ plaques. We also did not find any differences in total microglial number (Supplementary Fig. S2c) or gliosis (Supplementary Fig. S2d,e) in 5xFAD * *Psen1*^*KI/KI*^ mice compared to 5xFAD. Collectively, our data suggest that PS1 phosphorylation at serine 367 in microglia plays an important role in regulating Aβ metabolism in WT and 5xFAD mice.

**Fig. 5:**
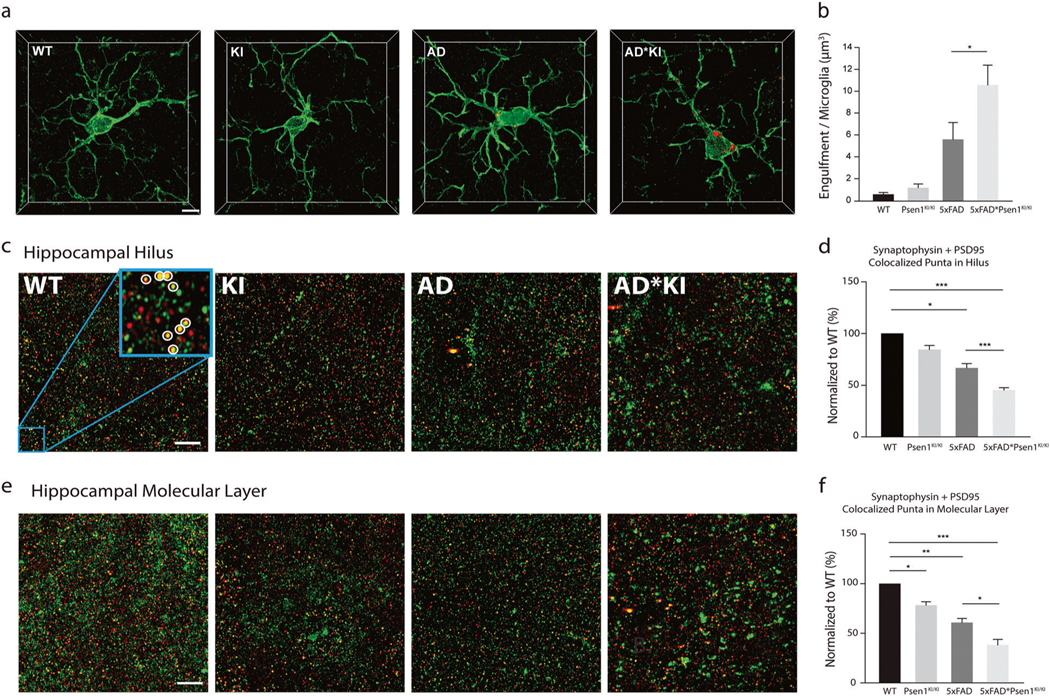
*Psen1*^*KI/KI*^ microglia accumulate more PSD-95 in an Alzheimer’s disease mouse model. **a** Accumulation of PSD-95 within microglia from mice hippocampus. Microglial volumes were generated based on IBA1 immunoreactivity (green). Scale bar: 5 μm. **b** Volumetric quantification of the PSD-95 inside microglia in wild-type, *Psen1*^*KI/KI*^, 5xFAD and 5xFAD*Psen1^*KI/KI*^ mice. **c** Representative z projection micrographs of the presynaptic marker synaptophysin (green) and the post-synaptic marker PSD95 (red) in the hippocampal hilus and molecular layer **e**. The micrographs correspond to the maximum intensity projection of 150 z-stacks across a 6 μm *z*-depth (see supplemental video 4 for 3D representation). Left to right: Wild-type (WT), *Psen1*^*KI/KI*^ (KI), 5xFAD (AD), and 5xFAD * *Psen1*^*KI/KI*^ (AD*KI) mice. A 4× higher magnification image in WT (blue box, 5 μm × 5 μm) was used to reveal the synaptophysin and PSD-95 spot pairs, as indicated within the white circle. Quantification of synapses in the hippocampal hilus (**d**) and molecular layer (**f**) as compared to their WT littermate controls. The 3D localization of presynaptic synaptophysin and postsynaptic PSD-95 puncta are identified using the “create spots” algorithm in Imaris (for details see Methods). Mice were 3 months old. Data are represented as mean ± SEM. *N* **=** 8–12 microglia per group, 3 mice per group. *p < 0.05, **p < 0.01, ***p < 0.001 using one-way ANOVA followed by Duncan’s Method.

Owning to the fact that the *Psen1*^*KI/KI*^ microglia displayed abnormal autophagy and inability to degrade Aβ, and deficient autophagy in microglia impairs neuronal spine pruning [49], we sought to investigate whether microglia containing *Psen1*^*KI/KI*^ would also accumulate more of the postsynaptic marker PSD-95. Our data revealed that *Psen1*^*KI/KI*^ * 5xFAD microglia retained more PSD-95 than the 5xFAD control microglia, (Fig. 5a, b). Next, we examined whether the AD mice brain containing *Psen1*^*KI/KI*^ microglia displayed abnormal synaptic density. We thus used super resolution structured illumination microscopy to quantify synaptic density in hippocampal molecular layer and hilus of WT, *Psen1*^*KI/KI*^, 5xFAD and 5xFAD*Psen1^*KI/KI*^ mice. Quantification of colocalized pre- and post-synaptic puncta (synaptophysin and post-synaptic density 95 (PSD95), for details see “Methods”) showed a significant loss of synapses in 5xFAD * *Psen1*^*KI/KI*^ mice compared to 5xFAD and a significant loss in *Psen1*^*KI/KI*^ mice compared to WT (Fig. 5c–f; Supplementary Fig. S3a).

## Discussion

Recently, we have shown that PS1, upon phosphorylation of serine 367 by CK1γ, can decrease Aβ levels through an autophagy-mediated mechanism involving the conversion of βCTF to Aβ independent of γ -secretase activity [20, 21, 50]. The present study adds an additional mechanism to our previous findings: we show that PS1 phosphorylation plays an important role in regulating autophagy-lysosomal pathway and Aβ levels by microglial cells. Collectively, our reports suggest that PS1 regulates Aβ levels at multiple levels.

In our current study we demonstrated that microglia from *Psen1*^*KI/KI*^ mice are functionally impaired. Using 2-photon intravital microscopy, we measured microglial response to a local injury. *Psen1*^*KI/KI*^ microglia displayed slower movement toward the site of injury and decreased formation of spherical-shaped inclusions. This spherical-shaped inclusion formation is attributable to a protective effect due to phagocytosis and removal of damaged tissue as well as shielding of the injured site [22, 48, 51]. Notably, microglia from aged mouse brain showed a decreased response to laser injury [52], similar to *Psen1*^*KI/KI*^ microglia. Interestingly, our RNA-seq data revealed pathways that are related to the ability of microglia to respond to a micro-injury in the brain. For example, phagosome formation and processing capacity plays an important role in microglial response to a micro injury in the brain [23]. Our data demonstrated that *Psen1*^*KI/KI*^ microglia display a significant accumulation of autophagosomes, inability to degrade autophagy substrates and clear AVs, which is potentially a result of defects in lysosome/autolysosome acidification in microglia. Although, we cannot rule out the possibility of a defect in the autophagosome-lysosome fusion, as we showed previously in neurons [20]. Our previous study in neurons, pointed to defects in autophagosome-lysosome fusion but not lysosome/autolysosome acidification [20]. Data suggest that autophagosome-lysosome fusion is not dependent on lysosomal acidification [53], which is in agreement with our previous report. Thus, disturbed lysosome/autolysosome acidification caused by *Psen1*^*KI/KI*^ might be microglial specific and disturbed autophagosome-lysosome fusion restricted to neurons. However, this remains to be confirmed. Our pathway analysis also revealed that 14–3-3 pathway was significantly altered in *Psen1*^*KI/KI*^ microglia. 14–3-3 proteins interact with microtubule-associated protein tau and induce tau phosphorylation [54]. Tau proteins regulate microtubule dynamics through microtubule-tau interaction [55]. Interestingly, it was shown that impairment of microtubule function due to tau hyperphosphorylation may promote dysregulation of the actin dynamics [56, 57]. Microglial shape changes rely on the rearrangement of the cytoskeletal proteins, in particular the actin microfilaments [58]. These cellular rearrangements in microglia are essential for microglia movement toward injury sites in the brain. Thus, it is possible that 14–3-3 proteins and Tau are mechanistically involved in *Psen1*^*KI/KI*^ microglia slower movement toward a site of laser micro-injury (Fig. 1a).

γ-Secretase activity regulates a multitude of signaling pathways and biological processes by influencing gene transcription via the processing of its substrates and consequently production of its intracellular domains (ICDs). Several ICDs produced by γ-secretase cleavage are known to translocate into the nucleus and regulate transcription. One of the most noted cases is the processing of the Notch receptor. Therefore, we considered that the alterations in gene transcription related to the lysosomal acidification induced by *Psen1*^*KI/KI*^ could be due to changes in γ-secretase activity. Indeed, we found that *Psen1*^*KI/KI*^ microglia show increased γ-secretase activity toward Notch. However, pharmacological inhibition of γ-secretase activity in microglial cells did not change *v-ATP6v0a1* mRNA levels or lysosomal pH in WT and *Psen1*^*KI/KI*^ microglia, discarding the potential involvement of γ-secretase activity in that process. Nonetheless, we investigated whether the decreased lysosomal *v-ATP6v0a1* mRNA and increased lysosomal pH in *Psen1*^*KI/KI*^ microglia was mediated by the transcriptional factor TFEB, a master regulator of lysosome biogenesis and autophagy, which overexpression has been shown to increase the levels of v-ATP6v0a1 [38]. Interestingly, *Psen1*−/− neural stem cells show reduced autophagosome formation and downregulated expression of autophagy–lysosome pathway, which seems to depend on TFEB [35]. Importantly, these effects did not depend on γ-secretase activity. Our data showed that TFEB mRNA levels is decreased in *Psen1*^*KI/KI*^ microglial cells compared to WT microglia. We next overexpressed an active form TFEB in WT and *Psen1*^*KI/KI*^ microglia and evaluate *v-Atp6v0a1* mRNA levels and lysosomal pH. Interestingly, active form of TFEB in *Psen1*^*KI/KI*^ microglia increased *v-Atp6v0a1* mRNA levels and normalized lysosomal pH compared to WT microglia. These results suggest that TFEB acts downstream to PS1 and regulates transcriptional changes related to v-atpases and consequently lysosomal acidification. The exact mechanism by which PS1 regulates TFEB remains to be elucidated. In our previous work we showed that PS1 phosphorylated at serine 367 binds Annexin A2, as opposed to non-phosphorylated *Psen1*^*KI/KI*^ [20]. Importantly, mutation of PS1 serine 366 or PS1 serine 368 did not affect the binding of Annexin A2 to PS1 [20]. Annexin A2 not only is known to translocate into the nucleus but it has been shown to form a complex with TFEB and YWHA/14–3-3 [59]. Thus, PS1 upon phosphorylation could potentially regulate TFEB through Annexin A2 independent of γ-secretase activity. Nonetheless, the molecular mechanisms by which PS1 regulates TFEB are incompletely understood. A recent report showed that cells deficient in PS1 display a significant reduction in TFEB-mediated clearance due to a reduction in Sestrin2 expression [36]. The authors then showed that PS1 deficient fibroblasts and iPSC-derived AD human neurons display impaired ability to initiate autophagy and reduction of the coordinated lysosomal expression and regulation network. Interestingly, the authors also showed an increase in Aβ 42/40 ratio in TFEB-deficient mouse brains.

In our present study we also demonstrated that microglia in 5xFAD * *Psen1*^*KI/KI*^ mice accumulate more post-synaptic protein PSD95 than 5xFAD mice in areas of the brain important for cognition. A recent work showed Aβ oligomers increase synaptic pruning by microglia and microglia can act as early mediators of synaptic loss in AD mouse models [60]. We observed a significant accumulation of PSD95 puncta in 5xFAD * *Psen1*^*KI/KI*^ microglia compared to 5xFAD, but not in *Psen1*^*KI/KI*^ compared to WT, possibly because endogenous mouse Aβ does not aggregated and accumulate in the mouse brain and therefore did not trigger microglial pruning. Moreover, our data showed that *Psen1*^*KI/KI*^ accelerated synaptic loss in an AD mouse model. Although part of these results can be attributable to spine pruning by microglia, it is possible that *Psen1*^*KI/KI*^ disturbs synapses in WT and 5xFAD mice by acting also directly in neurons independently of microglia.

Importantly, Semick et al. [61] reported that CK1γ2 gene is hypermethylated in vulnerable regions in the brain of sporadic AD patients, which results in lower CK1γ2 expression and could potentially lead to decreased phosphorylation of PS1 at serine 367. Elucidation of the mechanisms that regulate PS1 phosphorylation at serine 367 may aid in the development of therapies for AD. Due to the fact that PS1 serine 367 is preserved in humans, it would be extremely important to demonstrate that PS1 phosphorylation changes in human AD brains. However, technical challenges preclude such an evaluation. We and others have shown in several species that protein dephosphorylation occurs within seconds after death in animal models. Interventions not feasible in humans (microwave) are required to preserve protein phosphorylation. Future studies are needed to determine the extent to which abnormalities in microglia that contribute to AD are specifically mediated by microglial PS1.

## Methods

### Mice

C57BL/6 (000664), Cx3Cr1-GFP (005582), Psen1^loxP^ (004825), and 5xFAD (34848-JAX) mice were purchased from the Jackson Laboratories and maintained in our facilities. Psen1^S367A^ (*Psen1*^*KI/KI*^) constitutive knock-in C57BL/6J mice were generated by homologous recombination targeting exon 10. As wild type controls C57BL/6J, *Psen1*^*KI/KI*^ -negative littermates were used. *Psen1*^*KI/KI*^ mice was crossed with 5xFAD hemizygous mouse. 5xFAD hemizygous mouse * Psen1^KI/WT^ mice was then crossed with *Psen1*^KI/WT^ mice. 5xFAD hemizygous * *Psen1*^*KI/KI*^ mice were used in this study. Mice were weaned at the third postnatal week, genotyped by Transnetyx using real-time PCR and kept on a 12 h/12 h light/dark cycle (lights on at 7:00) with access to food and water ad libitum.

All mice were maintained at The Rockefeller University Animal facilities and used at 8–14 weeks of age for all experiments. Littermates of the same sex were randomly assigned to experimental groups. Both female and male mice were used for experiments, except for in vivo imaging where only males were used. Mice were anesthetized for all procedures involving potential pain/ stress. Animal care and experimentation were according to NIH guidelines and were approved by the Institutional Animal Care and Use Committee at The Rockefeller University (protocol #18035H).

### Primary cultures of mouse microglia and other treatments

Briefly, neonate (P0–P1) mouse brains cortices were dissociated in culture medium containing DMEM/F12 (ThermoFisher Scientific, 10565018) supplemented with 10% fetal bovine serum (ThermoFisher Scientific, 16000044) and 1% Penicillin–Streptomycin (ThermoFisher Scientific, 15140148). Cells were incubated in 75 cm^2^ flasks for 2 weeks at 37 °C, 5% CO_2_. Microglial cells were isolated according to Saura et al., (2003) [62]. For Aβ degradation experiments serum was omitted from the culture medium during the assay. Aβ oligomers were prepared according to Ledo et al., 2016 [63]. Primary microglial cultures were treated with 100 nM of Aβ 1–42 oligomers per 3 h. Aβ was detect using an ELISA kit (ThermoFisher Scientific, KHB3441). Aβ 1–42 peptide was purchased from AnaSpec (AS-24224). TFEB lentivirus containing a constitutively active version (S142A, S211A) of Tfeb was a gift from Dr. Jhimmy Talbot. Cells were transduced with an empty vector or TFEB-expressing lentivirus for 24 h, then medium was replaced, and cells were harvest after 48 h. TFEB protein levels was assessed by Western blot. DAPT treatment: cells were treated with vehicle or DAPT (10 μM) for 24 h. DAPT was purchased from Tocris /Bio-Techne (Cat. No. 2634).

PS1 deletion in primary microglia culture was performed according to Paolicelli et al. [64]. Psen1^loxP/loxP^ (JAX-004825) mice was used for primary microglia culture preparations. Psen1^loxP/loxP^ primary microglia culture was treated with recombinant TAT-CRE (100 U/mL medium, SCR508, EMD Millipore) to induce Psen1 gene deletion. Control Psen1^loxP/loxP^ microglia were treated with a solution containing 50% glycerol, 500 mM NaCl and 20 mM HEPES at pH 7.4.

### Aβ detection in brain extracts

Soluble Aβ was extracted using diethylamine (DEA) and insoluble Aβ was extracted with formic acid (FA). Aβ was detect using an ELISA kit (ThermoFisher Scientific, KHB3441).

Soluble Aβ: one brain hemisphere was homogenized in 850 μL cold tissue homogenization buffer (2 mM Tris pH7.4, 250 mM sucrose, 0.5 mM EDTA and 0.5 mM EGTA) containing protease inhibitors cocktail. After homogenization, 250 μL of a solution containing 0.2% DEA in 50 mM NaCl was added to the brain sample and centrifuged at 100,000 *g* for 1 h at 4 °C. Supernatant, which contains the soluble fraction, were treated with 0.5 M Tris HCl pH 6.8 (1/10 volume) and vortexed gently. Neutralized samples were analyzed by ELISA without further dilution or flash-frozen on dry ice and stored at −80 °C. Remained pellet was used for insoluble Aβ preparation, described below.

Insoluble Aβ: 125 μL cold formic acid (minimum 95%, Sigma, 5–0507) was added to the homogenate pellet (see above), then sonicated for 1 min on ice. Samples were then centrifuged at 109,000 × *g* for 1 h at 4 °C. After, 105 μL sample was diluted into 1.850 mL of room temperature FA neutralization solution (1 M Tris base, 0.5 M Na_2_HPO_4_, 0.05% NaN_3_), vortexed gently and stored at −80 °C. Samples were briefly mixed and incubated at 37 °C for 5 min prior to loading onto ELISA plates.

### Brain microglia isolation from adult mice and flow cytometry analysis

Mice were anesthetized with a Ketamine/Xylazine cocktail and perfused with 25 mL of Ca^2+^/Mg^2+^-free DPBS (Sigma). Brain was removed and placed in FACS buffer (PBS containing 5% FBS and 10 mM HEPES). Whole brains (except cerebellum) were minced with scissors and incubated with 4000 U/mL of collagenase D (Roche, 11088858001) at 37 °C for 30 min. Collagenase was inactivated by adding 10 mM EDTA for an additional 5-min incubation at 37 °C. Digested material was passed through a 70-μm cell strainer, followed by a centrifugation at 2000 rpm in 38% Percoll gradient for 30 min. Cell pellets were resuspended in FACS buffer and nonspecific binding to FC receptors was blocked by incubation with a CD16- and CD32-specific antibody (BD-Pharmingen 553141) for 15 min. Cells were then washed and stained with the markers described below to certify cell population specificity.

Cx3cr1^+^ – CD45^+^ – Csf1r^+^ – Cd11b^+^ – Ly-6c (negative). Fluorescent-dye-conjugated antibodies were purchased from Biolegend (anti-CD115 (Csfr1), 135510; anti-F4/80, 123131; anti-Cx3Cr1, 149016; anti-CD117 (c-kit), 105824; anti-Ly6c, 128033), Invitrogen (Anti-CD11b, 47–0112-82; anti-CD45, 56–0451-82) or BD-Pharmingen (anti-CD16/CD32 (FC blocking); 553141). Live cells were verified using DAPI (4′,6-Diamidino-2-Phenylindole, Dilactate), (D3571, Thermofisher). Flow cytometry data were acquired on an LSR-II flow cytometer (Becton Dickinson) and analyzed using FlowJo software (Tree Star).

### RNA isolation from primary microglial cultures

RNA was isolated using the ReliaPrep™ RNA Miniprep Systems (Promega, Z6011). Briefly, cells were washed with ice-cold sterile 1X PBS. After, 250 μL of BL + TG Buffer (provided by the manufacturer) was added to the cells. Lysate was then transferred to a 1.5 mL tube with 85 μL of isopropanol and mixed by vortexing for 5 s. After, lysate was transferred to a ReliaPrep™ minicolumn and the following steps were conducted according to the instructions provided by the manufacturer.

### RNA sequencing and bioinformatics

#### Adult mouse microglia:

20,000–35,000 cells per each sample were sorted directly into RNA lysis buffer (Qiagen, 79216) supplemented with 2 M dithiothreitol. RNA was isolated using RNAeasy plus micro kit (Qiagen, 74034). Briefly, homogenized lysate was transferred to a gDNA eliminator spin column placed in a 2 mL collection tube (supplied by the manufacturer). The following steps were conducted according to the instructions provided by the manufacturer. For all RNA samples, RNA integrity number (RIN) was ≥8.5. After RNA isolation, 1 ng of total RNA was used to generate full length cDNA using Clontech’s SMART-Seq v4 Ultra Low Input RNA Kit (634888). cDNA was then used to prepare libraries using Illumina Nextera XT DNA sample preparation kit (FC-131–1024). Libraries with unique barcodes were pooled at equal molar ratios and sequenced on Illumina NextSeq 500 sequencer to generate 150 bp single reads, following manufacturer’s protocol.

The reads were aligned using the STAR version 2.3.0 software that permits unique alignments to Mouse Ensembl genes. Differential expression was determined using edgeR software with default settings. Expression is given in Counts per million (CPM).

#### Microglia identity signature genes:

defined as 239 genes that are specifically expressed in mouse microglia compared to monocytes and other immune cell types [26].

### Real-time relative quantification PCR

qPCR was performed using Taqman reagents. Rpl23 or ActB were used to normalize samples. Predesigned probes used were purchased from IDTDNA. mRNA levels are expressed using the 2^−ΔΔCt^ method [65].

### Exo-cell γ-secretase activity assay

Cells were seeded in 96-well culture plates. Media was then removed, and cells were washed with PBS. Cleavage assay mixture included a final concentration of PIPES Buffer (50 mM PIPES, pH 7.0, 150 mM KCl, 5 mM CaCl_2_, 5 mM MgCl_2_), 0.25% CHAPSO detergent, protease inhibitor cocktail, Notch substrate (0.4 μM), and 0.1% DMSO or JC2 at 1 μM final concentration. Cleavage assay was performed at 37 °C for 2.5 h. Product of Notch cleavage was recognized by AlphaLISA detection comprised of anti-activated Notch antibody SM320, protein A-conjugated acceptor beads, and streptavidin-conjugated donor beads (PerkinElmer) [66]. Activity readout was expressed as arbitrary units minus the background signal from γ-secretase inhibitor sample and normalized to protein concentration.

### Western blot

FACS sorted microglial cells derived from WT and *Psen1*^*KI/KI*^ mice were lysed in a buffer containing 2% CHAPSO, 50 mM Hepes pH 7.4, 150 mM NaCl, protease inhibitor cocktail (PhosStop, Sigma-Aldrich) and protease inhibitor cocktail mini (Roche). PS1 was immunoprecipitated by incubating cell extracts overnight at 4C with anti-PS1 antibody (Mab5232, Sigma-Aldrich) covalently bound to agarose beads, then run in a Tricine SDS-PAGE and blotted with anti-PS1-pS367 antibody. The membrane was stripped, and reblotted with anti- total PS1 (Mab5232, Sigma-Aldrich). GAPDH antibody was from GeneTex (Cat No. GTX89740).

### Immunohistochemistry

Animals were anesthetized and perfused with PBS, followed by 4% formaldehyde in PBS. Fixed brains were removed and cryoprotected in increasing concentrations of sucrose up to 30% w/v in PBS. Brains were frozen on dry ice in OCT, and 50 μm or 150 μm coronal sections were made on a cryostat and stored in section freezing solution at −20 °C. Groups of three sections per animal (three animals per condition in each experiment) were stained with the following procedure: wash 10 min in PBS, permeabilization 20 min with PBS + 0.2% Triton x-100 (PBST), block 40 min with 10% NGS in PBST, incubate primary antibody overnight in PBST with 5% NGS, wash 3 × 10 min in PBST, incubate 2 h in secondary antibody in PBST with 5% NGS, wash in 3 × 10 min in PBS, and mount in CFM-2 mounting medium. For thicker sections, additional permeabilization was performed by 10 min incubation steps with increasing methanol in PBS (25%, 50%, 75%, 100%, 75%, 50%, 25%), and primary antibody staining was performed at RT overnight. All incubation steps were performed with gentle agitation on a nutating mixer. Microglia (Iba1), post-synaptic marker PSD95 and Aβ were stained using antibodies from Abcam (anti-Iba1, ab5076, 1:25; antiPSD95, ab76115, 1:500) and Biolegend (anti-Aβ, clone 6E10, 803003, 1:500), respectively. Sections were imaged on a Zeiss LSM 710 with a ×40, ×63 oil or ×100 oil immersion objective. All primary and secondary antibodies were validated before use. Secondary antibody only control was performed and resulted in no false positives or non-specific binding (data not shown).

### LC3-GFP expression and immuno-electron microscopy

For LC3-GFP expression, LentiBriteTM Lentiviral Biosensor (Millipore, 17–10193) was used. Briefly, lentiviruses were incubated in the cell medium at 40 multiplicity of infection for 24 h, and then medium was replaced every 24 h for 48 h. After treatment, cells were subjected to high pressure freezing (Leica EMPAC2) and freeze substitute in 0.2% uranyl acetate in 95% acetone and 5% water. Subsequently they were embedded in Lowicryl HM20 (Electron Microcopy Sciences, 14340) at −40 °C and cut into ultrathin sections. Next, ultrathin sections were incubated with 3% BSA (Sigma-Aldrich, A7906) and 0.1% saponin (Sigma-Aldrich, 47036), 0.1% cold fish skin gelatin (Sigma-Aldrich, G7765) in 20 mM Tris buffer saline (pH 7.4) for 2 h at RT, an anti-GFP raised in chicken (1:300) (Aves Lab Inc, GFP-1020) at 4 °C overnight. Antigen-antibody complexes were recognized by anti-chicken colloidal gold tagged with 12 nm colloidal gold (Jackson immuno Research Lab Inc, 703–205-155) by 2 h incubation at RT. Negative control was done with the same procedure, except for omitting the primary antibody incubation. For autophagosomes in primary microglial cell cultures magnification was ×6600. For LC3 staining in primary microglial cell cultures magnifications was ×8300. For autophagosomes in microglia from adult mice brains magnification was ×10,000. Immuno complexes were examined under a JEOL JEM 100CX transmission electron microscope with the digital imaging system (Advantage Microscopy Technology Corp, XR41-C) in the Electron Microscopy Resource Center in The Rockefeller University.

### Lysosomal pH

Primary microglial cells from neonate mouse were plated on a clear bottom 96-well plate with black walls and incubated with 500 μg/mL of the acidotropic probe, Lysosensor yellow/blue dextran (Invitrogen, L22460) for 24 h. After, cells were washed with PBS and fluorescence was detected using a microplate reader with an excitation of 340 nm and an emission wavelength of 430 and 535 nm. Relative lysosomal pH levels were determined by the emission ratio of 430/535 nm.

### Cranial window technique for two-photon in vivo imaging

Mice received one injection of 100 μL of Ketamine/Xylazine cocktail (40 mg/mL ketamine 6 mg/mL Xylazine) (Sigma, K113) intraperitoneally, followed one hour later by intraperitoneal injection of 0.8 g/kg of Urethane (Sigma, U2500). A circular area of 2–2.5 mm diameter was drawn in the skull and thinned with a sterile 1RF 007 drill bit using a Microtorque II drill set (Ram Products Inc, 10145FT) at ~4000 rpm. The skull piece was then carefully removed with forceps to prevent agitation of the tissue and rupture of superficial blood vessels. A small drop of artificial CSF was placed on the tissue immediately after removing the skull and a 5 mm coverslip was placed to cover the exposed tissue. A small drop of Permabond^®^ glue was carefully applied under the coverslip edged to affix the coverslip to the skull. After the glue was dried and the coverslip was attached to the skull, a custom-made metal plate was glued to the skull using a low viscosity silicone (World precision Instruments, KWIK-SIL). The concave plate was designed to hold immersion fluid (water) for the dipping lens. The mouse was then transferred to a custom-made platform to prevent any motion of the head during imaging.

Multiphoton microscopy experiments were performed according to Nimmerjahn et al. with minor modifications [22]. In vivo image acquisition was performed on an Olympus FV1000MPE Twin upright BX61 multiphoton system with a 25×/1.05 N.A. Plan objective. A lesion was introduced at a confined area (spot size of ~10 μm laterally in a single plane) using elevated illumination intensities of a 473 nm LD laser at 50% power for 30 s at a typical depth from the cortex surface of 85 μm. Immediately preceding the lesion, image stacks were acquired using a coherent Chameleon Vision II IR laser at 900 nm, covering a depth range of ±40 μm from the lesion site. Stack volumes were taken at a frequency of 0.61 volumes per minute and with an axial resolution of 1 μm. Microglia encroachment analysis was performed by measuring the cumulative intensity of pixels within a fixed volume centered at the lesion site after subtraction of the initial intensity. Image analysis was done blind with regard to experimental condition. Image stacks and time series were analyzed using ImageJ software from the National Institutes of Health.

### Morphology tracing and volume images

Cell tracing and volume image rendering were performed using Bitplane’s Imaris software. Microglia morphology was characterized using the filament tracing tool. Manual correction of the autopath tracing was performed on 100 μm confocal stack images of Iba1 staining in the cortex. For tracing experiments, six hemispheres were used from three different mice for each genotype. Volume images from confocal or light sheet microscope stacks are all maximum intensity projection images.

### iDISCO visualization and ClearMap quantitation of plaques

Intact brain hemispheres were stained for Aβ plaques using a previously described protocol for iDISCO [47]. Quantification of Aβ plaques was performed with the previously published ClearMap tool [47, 67]. ClearMap automates whole hemisphere light sheet image registration to the Allen Brain Atlas and detects and quantifies objects in all annotated brain regions. Detection settings were as follows: background subtraction (7 pixels), difference of Gaussian filter (5, 5, 9), extended maxima h-max (20) and size (5 pixels) and cell shape threshold (200). Output values of ClearMap are number and intensity of objects detected.

### Airyscan confocal super-resolution microscopy and quantitative analysis

Brain slides were imaged on an inverted Zeiss LSM 880 laser-scanning confocal microscope equipped with an Airyscan super-resolution module (Carl Zeiss AG, Germany), which improves optical resolution in all spatial directions by 1.7-fold [68, 69]. All images were acquired using a 63 × 1.4 NA oil objective lens (Plan-Apochromat, 1.40 Oil DIC M27), with the pinhole set at 1.25 AU. For imaging microglia engulfment, single microglia was centered in the image frame and the Z-stack dimensions were set manually by tracking IBA-1 labeled processes, which were typically ranged from 20 to 30 μm for a given microglia. Images were acquired as 1164 × 1164 pixels (zoom factor = 2.7) with a Z-interval of 0.1 μm, corresponding to a voxel dimension of × 0.04 × 0.1 μm in *x*, *y*, and *z* directions. For imaging synaptic density, Z-stack dimensions were set to 10–20 μm. Images were acquired as 868 × 868 pixels (zoom factor = 3.6) with a Z-interval of 0.04 μm, corresponding to a voxel dimension of 0.04 × 0.04 × 0.04 μm in *x*, *y*, and *z* directions. To reduce overall photobleaching and fluorescence crosstalk, maximum of two channels were defined and then scanned simultaneously using MBS 488/561 as the dichroic beam splitter. Airyscan processing was done in 3D mode at default settings using Zeiss Zen 2 software (black edition).

### Quantification of synaptic density and microglia engulfment

Image analysis was performed as previously described by Schafer et al., 2012 and Hong et al. [60, 70] with minor modifications. In brief, Airyscan confocal z-stacks were imported into Imaris® (version 9.1.2) software (Bitplane Inc.) and target of interest was segmented out from rest of the 3D dataset by manually selecting in each z-stack. For experiments regarding the engulfment of PSD95 and Aβ by microglia each 15 μm z-stack contains only one microglia was cut, collected in Imaris and the volumetric density calculated. PSD95 and Aβ fluorescence signal outside of the microglial surface was discarded to ensure that only engulfed PSD95 and Aβ was calculated. For analysis of the co-localization of pre-synaptic marker synaptophysin and the post-synaptic marker PSD95, each 6 μm excluding very empty regions was collected in Imaris, then segmentation of spots in both fluorescence channels was performed separately, followed by quantification the number of colocalized spots (≤200 nm distance between spot centers of two synaptic channels). Representative images (Fig. 5c, e) were the maximum intensity projection of the Z-stack images (across 6 μm) which makes larger aggregates more noticeable. However, the actual detected synapses (colocalized spots) are fairly similar in terms of size, regardless of different samples and their depth in the tissue.

### Statistical analysis

All results are presented as mean ± SEM. Prism software or Matlab were used for data analysis, except for synapse puncta quantification, SigmaPlot 13.0, Systat Software was used. No statistical methods were used to predetermine sample sizes, sample sizes were determined according to data reported in previous publications. Normality tests and *F* tests for equality of variance were performed before choosing the statistical test. Unless otherwise indicated, two-tailed *t*-test or one-way analysis of variance with Dunnett’s multiple comparison test was used. *P* < 0.05 was considered significant (*p < 0.05, **p < 0.01, ***p < 0.001, ****p < 0.0001). Animals in the same litter were randomly assigned to different experimental groups and blinded to experimenters.

## Supplementary Material

Video 3

Video 4

Video 1

Video 2

Supp 1

Supp 2

Supp 3

Supp 4

Data 1
