# Supplementary material for: Presenilin 1 phosphorylation regulates amyloid-β degradation by microglia
Source: Mol Psychiatry. 2020 Aug 13;26(10):5620–35. doi: 10.1038/s41380-020-0856-8 (PMC7881060; doi:10.1038/s41380-020-0856-8)
Supplement: Supplementary file 5 — Supplemental Material [file 41380_2020_856_MOESM5_ESM.pdf]

Presenilin 1 phosphorylation regulates amyloid-β degradation by microglia

Jose Henrique Ledo[1 #], Thomas Liebmann[1], Ran Zhang [2], Jerry C. Chang [1], Estefania P. Azevedo [3], Eitan Wong [4], Hernandez M. Silva [5], Olga G Troyanskaya [3, 6], Victor Bustos[1], Paul Greengard [1†].

[1] Laboratory of Molecular and Cellular Neuroscience, The Rockefeller University, New York, New York, 10065, USA.

[2] Lewis Sigler Institute for Integrative Genomics, Princeton University, Princeton, New Jersey, 08544, USA.

[3] Laboratory of Molecular Genetics, The Rockefeller University, New York, New York, 10065, USA.

[4] Chemical Biology Program, Memorial Sloan Kettering Cancer Center, New York, New York, 10065, USA.

[5] Skirball Institute of Biomolecular Medicine, New York University School of Medicine, New York, New York, 10016, USA.

[6] Flatiron Institute, Simons Foundation, New York, New York, 10010, USA.

[†] In Memoriam

# Correspondence should be addressed to: Jose H. Ledo – jledo@rockefeller.edu

Supplemental Figures Legends

Supplementary Figure 1: Lack of phosphorylation at serine 367 in PS1$^{KI/KI}$ microglia. Number of microglial in *Psen1* $^{KI/KI}$ mouse. FACS/Sorting gating strategy for isolating microglia from mouse brain.

(A) Total levels of PS1 in microglial cells from WT or *Psen1*$^{KI/KI}$ mice was analyzed by Western blot. GAPDH was used as loading control. (B) Phosphorylation of PS1 at serine 367 in microglial cells from WT or *Psen1*$^{KI/KI}$ mice was analyzed by Western blot using pS367-PS1 antibody. Total PS1 was immunoprecipitated from microglia lysates using an anti-PS1 antibody covalently bound to agarose beads, and immunoblotted with anti-PS1 pS367, stripped and reblotted against total PS1. (C) Schematic diagram showing the strategy used to verify microglial branching in Figure 1. Each segment is represented in different color (1-4), terminal endings are represented as number 4. (D) Confocal stack of microglia in the 2-month-old WT or Psen1$^{KI/KI}$ mouse brain. Scale bar represents 50 µm. Quantification of microglial density (cells / 10^{-3} mm^3), data represent means ± SEM (*n* = 5 mice per group). (E) Microglial cells were harvested from WT or *Psen1*$^{KI/KI}$ mice and gated for CD45$^+$, CD11B$^+$, CX3CR1$^+$, CSF1R$^+$, C-KIT$^-$, LY-6C$^-$. N = 3-4 individual brains per group. (E) Quantitative RT-PCR performed for *Psen1* (left panel) and *Atp6v0a1* (middle panel) in control or *Psen1*^{KO} primary microglia culture (E) Lysosomal pH of control or *Psen1*^{KO} primary microglia culture was determined using LysoSensor Yellow/Blue dextran (450/535 nm) (right panel). Data represent means ± SEM (*n* = 5 independent cell culture preparations; *****P* < 0.0001, Student's t-test).

Supplementary Figure 2: Aβ levels, γ-secretase activity and gliosis in 5xFAD and 5xFAD* *Psen1* $^{KI/KI}$ mice.

(A) The levels of soluble Aβ40 or Aβ42 and insoluble Aβ40 or Aβ42 in 3-month-old mouse brains were analyzed by ELISA. (B) Exo-cell γ-secretase activity assay for recombinant APP substrate in 5xFAD or Psen1$^{KI/KI}$ mouse. γ-Secretase activity is expressed as arbitrary units (for details see Methods). (C) Quantification of microglial density in 5xFAD and 5xFAD* *Psen1* $^{KI/KI}$ mice (cells / 10^{-3} mm^3). (D) Confocal stack of astrocytes (GFAP yellow) or (E) microglia (Iba1 cyan, Aβ plaque magenta, DNA yellow) in the 3-month-old mice brains. Scale bar represents 10 µm. Quantification of GFAP+ cells (D right panel) or Iba-1 intensity (E right panel) in 5xFAD and

5xFAD* *Psen1* $^{KI/KI}$ mice using fluorescence intensity values. Data represent means ± SEM (n = 5 mice per group; **$P < 0.01$ ***$P < 0.001$, Student's t-test).

Supplementary Figure 3: Quantification of synaptophysin and PSD95 in WT, *Psen1* $^{KI/KI}$, 5xFAD and 5xFAD* *Psen1* $^{KI/KI}$ mice.

(A) Quantification of synaptophysin and PSD95 in the hippocampal hilus and molecular layer related to Figure 5 (details described in methods). Mice were 3 months old. Data are represented as mean ± SEM. N = 8-12 microglia per group, 3 mice per group. *$P < 0.05$, **$P < 0.01$, ***$P < 0.001$, one-way ANOVA followed by Duncan's Method.

Supplemental Table S1 content:

- Gene expression of adult microglia, Related to Figure 2.

- Quantification of Aβ plaques using ClearMap, Related to Figure 4.

Supplemental movie legends

Supplemental movie 1: WT versus *Psen1* $^{KI/KI}$ microglia activation upon injury using a highly localized laser-induced micro lesion, Related to Figure 1 A-B.

After the micro lesion *Psen1* $^{KI/KI}$ microglial cells displayed a slower kinetic response compared to WT. For details see Methods section: Multiphoton microscopy.

Supplemental movie 2: Three-dimensional reconstruction used for the analysis of microglial morphology, Related to Figure 1 C-E.

For details see Methods section: Morphology tracing and volume images

Supplemental movie 3: Confocal imaging of amyloid-β plaques using iDISCO, Related to Figure 4 D.

Whole hemisphere labeled for amyloid-β plaques (Congo red) with Confocal Microscopy using iDISCO. For details see Methods section: iDISCO visualization and ClearMap quantitation of plaques.

Supplemental movie 4: Three-dimensional reconstruction used for the analysis of synapses (synaptophysin – green and PSD95- red), Related to Figure 5 C-F.
