# Supplementary figures and images for: Presenilin 1 phosphorylation regulates amyloid-β degradation by microglia
Source: Mol Psychiatry. 2020 Aug 13;26(10):5620–35. doi: 10.1038/s41380-020-0856-8 (PMC7881060; doi:10.1038/s41380-020-0856-8)

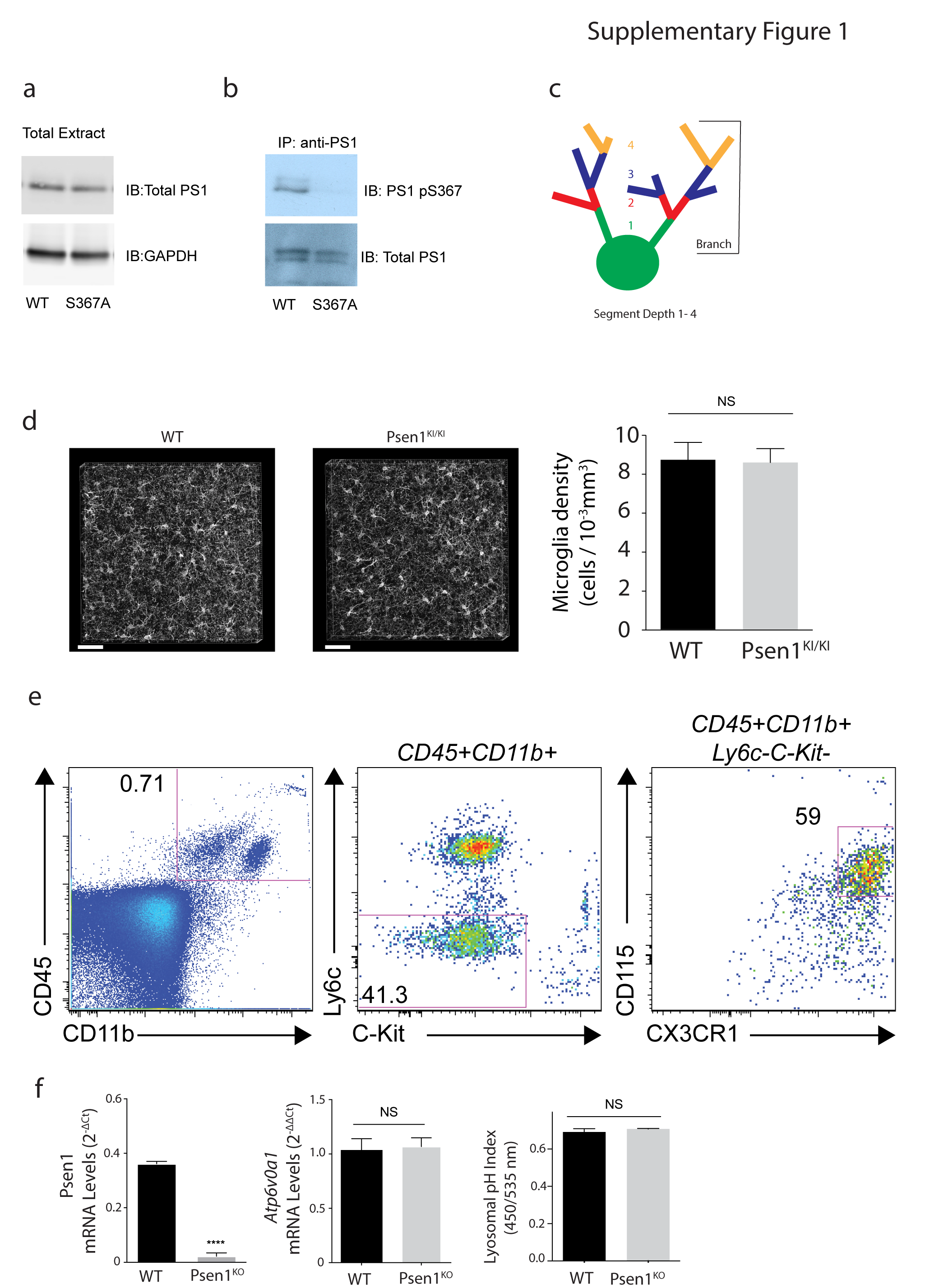

Supplement: Supplementary file 7 — Supplementary Data 1 [file 41380_2020_856_MOESM7_ESM.tif]

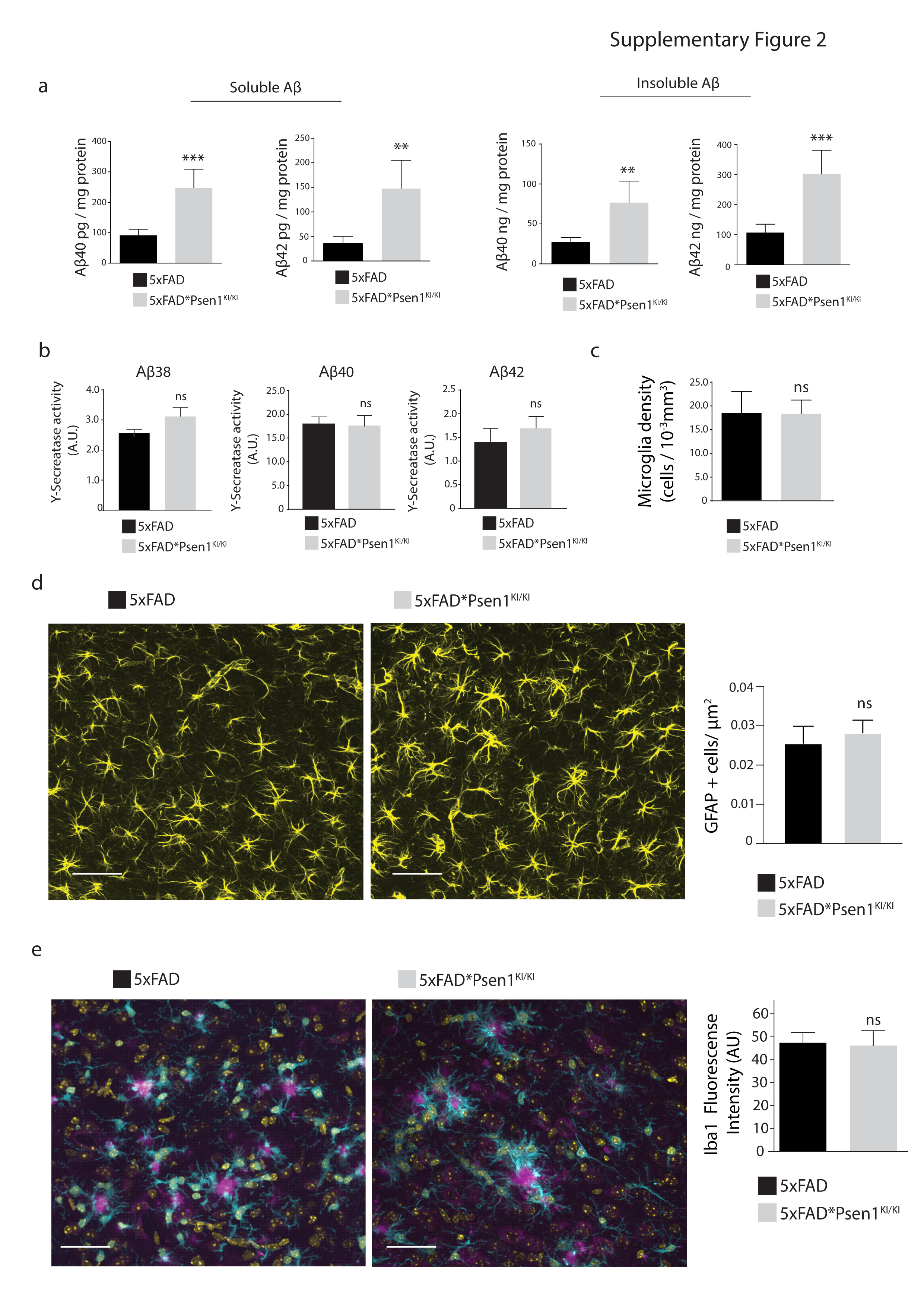

Supplement: Supplementary file 8 — Supplementary Data 2 [file 41380_2020_856_MOESM8_ESM.tif]

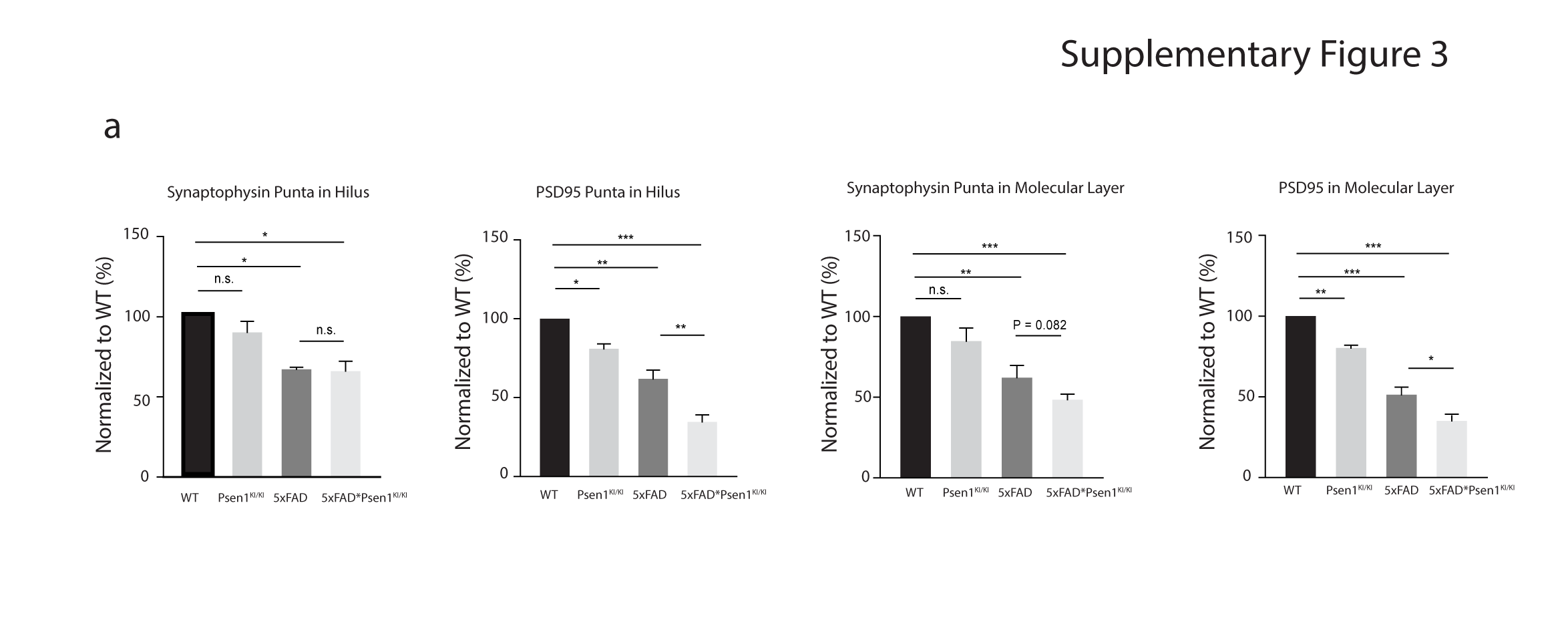

Supplement: Supplementary file 9 — Supplementary Data 3 [file 41380_2020_856_MOESM9_ESM.tif]
